# The importance of being relevant: modulation of magnitude representations

**DOI:** 10.3389/fpsyg.2013.00369

**Published:** 2013-06-26

**Authors:** Tali Leibovich, Liana Diesendruck, Orly Rubinsten, Avishai Henik

**Affiliations:** ^1^The Cognitive Neuropsychology Laboratory, Department of Cognitive Sciences, Ben-Gurion University of the NegevBeer-Sheva, Israel; ^2^The Cognitive Neuropsychology Laboratory, Department of Psychology and the Zlotowski Center for Neuroscience, Ben-Gurion University of the NegevBeer-Sheva, Israel; ^3^Department of Computer Sciences, Ben-Gurion University of the NegevBeer-Sheva, Israel; ^4^Department of Learning Disabilities, University of HaifaHaifa, Israel

**Keywords:** numerical cognition, ratio, Weber's law, comparative judgment, numerical Stroop

## Abstract

The current study aims to answer two main questions. First, is there a difference between the representations of the numerical and the physical properties of visually presented numbers? Second, can the relevancy of the dimension change its representation? In a numerical Stroop task, participants were asked to indicate either the physically or the numerically larger value of two digits. The ratio between the physical sizes and the numerical values changed orthogonally from 0.1 (the largest difference) to 0.8. Reaction times (RT) were plotted as a function of both physical and numerical ratios. Trend analysis revealed that while the numerical dimension followed Weber's law regardless of task demands, the physical ratio deviated from linearity. Our results suggest that discrete and continuous magnitudes are represented by different yet interactive systems rather than by a shared representation.

## Introduction

By a very early age, we estimate and compare discrete (number of items in a group) and continuous (brightness, loudness, size, etc.) magnitudes. This ability is important for survival across species. Honeybees distinguish between flowers by “counting” the numbers of petals (Leppik, 1956), lions assess the number of their opponents by listening to their roars, and act accordingly in order to survive (McComb et al., 1994). There are many other examples [e.g., fish (Agrillo et al., 2008); birds (Koehler, 1951); avian (Lyon, 2003); amphibians (Uller et al., 2003)].

How different magnitudes are represented and processed is one of the pressing questions in numerical cognition literature. A recent theory—the *approximate number system* (ANS) (Cantlon et al., 2009)—emphasizes the commonalities between discrete and continuous magnitudes (numbers, numerosity, time, physical size, brightness, etc.) and suggests that all these magnitudes are processed by a common algorithm. One of the hallmarks of the ANS is that performance in comparative judgments of the investigated magnitudes is best described by Weber's law. Namely, the ability to discriminate between two magnitudes depends on the ratio between them (the ratio effect). This ratio dependency is also the distinguishing feature of *core system 1* suggested by Feigenson et al. (2004). *Core system 1* is a system that represents approximate numerical magnitudes independently from non-numerical properties. Note, however, that *core system 1* relates only to non-symbolic quantities. Walsh (2003) suggested that all magnitudes, in all modalities, are represented in the same region in the brain—the parietal lobe. Moreover, he argues that since they are all needed to allow us to physically interact with the environment, the purpose of all magnitude processing is to guide motor actions.

Numbers are special magnitudes; they are symbols of size. No other species has developed this kind of representation. Dehaene and Akhavein (1995) suggested that numbers are a type of special language. According to this view, we share with other animals the “number sense”—a system that enables us to crudely estimate quantities. Alongside the “number sense,” which allows for an approximate representation of numbers, exists a symbolic representation in the form of numbers that enables us to accurately represent magnitudes. Several findings support that claim. Studies conducted with adults from secluded Amazonian tribes with a very limited numerical lexicon revealed that the representation of magnitudes is less accurate than that of participants from the West of the same age (Gordon, 2004; Pica et al., 2004). However, other cultural differences may contribute to these differences; in cultures with a limited numerical lexicon, children are not taught mathematics systematically—for example, they are not exposed to linear representations of numbers (such as on a ruler).

Izard and Dehaene's (2008) study provides additional evidence that the semantic meaning of numbers contributes to an exact representation of magnitudes. In this study participants were briefly exposed to arrays of dots and had to estimate their numerosity. One group of participants was first introduced to a standard array and told that it was made up of 30 dots. Magnitude estimations made by this group were more accurate than the group that was not introduced to a standard. The authors concluded that the verbal numerical value given for the standard “calibrated” the mental number line. In order for that manipulation to take effect, one must understand the meaning of the number. For example, introducing a standard of 500 dots to a child that can count only up to 100 does not create the same calibration effect.

In light of these differences, Cohen Kadosh et al. (2005) reviewed studies that searched for the commonalities and differences between numbers and other magnitudes, such as physical size, time, and brightness. Behaviorally, a representation is considered to be shared among different magnitudes if these magnitudes are characterized by similar effects, specifically, the distance and size effects. The distance effect was first discovered for numbers by Moyer and Landauer (1967). The authors presented adult participants with pairs of numbers, different numerical distances apart, and asked them to indicate the numerically larger number. The numerical distance was found to modulate reaction times (RT). Namely, RT was faster as the distance between the numbers grew (e.g., faster response for [2 8] than for [6 8])—the distance effect. In addition, the size of the numbers affected RT; for a constant distance, RT for two small numbers (e.g., 2 3) was faster than for two large numbers (e.g., 7 8)—the size effect. Note that while the distance effect alone explains a significant part of the variance, the ratio (i.e., smaller divided by larger magnitude) explains more variance in number comparisons (Moyer and Landauer, 1967). Similar effects were found for comparison of physical sizes such as line length, brightness, and angles (Cohen Kadosh et al., 2005).

Another way to approach the question of shared representations is by looking at interactions when comparing two different dimensions. In a study by Henik and Tzelgov (1982), participants were presented with two digits and were asked to indicate the larger number (with respect to either physical size or numerical value). In congruent trials, physically larger numbers were also numerically larger (e.g., 4 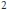). In incongruent trials, physically larger numbers were numerically smaller (e.g., 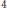 2). In neutral trials, only one dimension was manipulated. In a physical task, neutral trials included the same number in two different physical sizes (e.g., 2 2), and in a numerical task, neutral trials included two different numbers of the same physical size (e.g., 2 4). Responses were influenced by the degree of congruency between relevant and irrelevant dimensions (congruent trials were faster than neutrals, and incongruent trials were the slowest), suggesting automatic processing of numerical values, even when these values were irrelevant to the task: the size-congruity effect. Moreover, manipulating the numerical distance had an effect even when it was irrelevant to the task. Hence, numerical distances were automatically computed in this task and they affected relevant judgment. The size-congruity effect was found with other continuous magnitudes such as brightness (Cohen Kadosh et al., 2008a) and the height of the number (Rubinsten and Henik, 2005). According to Cohen Kadosh et al. (2005), the size-congruity effect “suggests that different types of magnitude tap the same magnitude mechanism” (p. 1283).

To summarize, in the current literature, the presence of size and distance effects and the size-congruity effect in different magnitudes is brought as evidence of a shared representation. We propose that although comparative judgment of different dimensions results in a ratio effect, there might be subtler differences indicating that these magnitudes are processed by different systems. We shall now explain that suggestion.

Some studies in numerical cognition literature have concluded that ratio dependency is evidence of compliance with Weber's law (see Odic et al., 2013). However, Weber's law is concerned with *linear ratio dependency*. Thus, performance for a specific magnitude might be described as ratio-dependent but non-linear. Why is that distinction important? Plotting RT as a function of the ratio between two magnitudes [smaller divided by larger; see Cantlon and Brannon (2006)] suggests that for a fixed-size ratio increment of *X*, RT will increase by a constant amount. Thus, the difference between responses to ratios 0.2 and 0.3 is identical to the difference between responses to ratios 0.7 and 0.8. Namely, discriminability increases linearly, yielding a linear trend. In contrast, a non-linear ratio dependency, which can be described by a power function (e.g., *Y* = *ax*^*b*^ + *c* with *b* > 1), would suggest that RT does not change by a constant amount. RT increases, non-linearly, with the similarity (of size, for example) between the stimuli. For example, an increment from ratio 0.2 to 0.3 produces a lower increase in RT than an increment in size ratio from 0.7 to 0.8. This would imply that discriminability becomes more difficult with increase in similarity.

## The current study

In the current study, participants were presented with two numbers and were asked to choose either the physically or the numerically larger number. The ratio between the magnitudes (smaller divided by larger magnitude; for physical size or numerical value) varied from 0.1 to 0.8, with 0.1 being the largest difference (e.g., 2 and 7 or 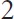 2), and 0.8 being the smallest difference (e.g., 6 and 8 or 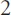 2). Next, we plotted RT as a function of the magnitude ratio for every task. In that way, we could examine more closely possible differences between performance in comparison of numerical values and physical sizes. In Experiment 1, only one dimension was manipulated [i.e., the neutral condition in a numerical Stroop task as described in Henik and Tzelgov (1982)]. For every participant and for every task, RT was plotted as a function of the ratio between the to-be-compared magnitudes. These plots were compared once to a power function (*RT* = *ax*^*b*^ + *c*) and once to a linear function, and the fit values (*r*^2^) were recorded. If the fit to the power function when *b* = 1 and the fit to the linear function did not differ, it indicated that the trend was linear. However, if the fit to the power function when *b* ≠ 1 was better than the fit to the linear function, then the trend deviated from linearity. Note that the exponent is not just one more free parameter that can explain more variance, because if the trend is linear, the exponent *b* = 1 would force the power function to be linear as well. Thus, the exponent can only add to the explained variance if different than 1.

Based on previous findings, we expected that performance in the numerical task would be best described by Weber's law; first, Weber's law has been suggested in studies that examined numerical discrimination (Moyer and Landauer, 1967; Cantlon and Brannon, 2006), and second, as suggested by Izard and Dehaene (2008), the exact numerical values allow us to represent magnitudes more accurately. There is no dispute about the value of the number 3, or that the difference between 9 and 4 is exactly 5. This is true for all the number pairs used here (i.e., numbers from 1 to 9). Thus, it is reasonable to believe that the level of difficulty will increase with increase in similarity between the stimuli^1^.

Unlike the exact verbal representation of the difference between two numbers (i.e., 3 and 5), we cannot tell the exact *physical* size of the number 3 and we cannot tell the exact difference in size between 3 and 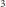. For that reason, we might expect deviations from Weber's law. In addition, representation of the mental number line was found to be modulated by other factors, such as attention. For example, Anobile et al. (2011) found that under attentionally demanding conditions, an otherwise linear mapping becomes compressed and non-linear. It is possible that the verbal aspect of numbers has a similar effect on the representation of their physical size. Accordingly, for the physical task, we asked whether performance in the physical comparison task would comply with Weber's law or not.

In Experiment 2, both numerical and physical dimensions were orthogonally manipulated to create congruent and incongruent conditions [as described in Henik and Tzelgov (1982)]. RT was plotted as a function of both the physical and the numerical magnitudes. Here we investigated if (and how) the relevancy of the dimension influences performance. Assuming that physical and numerical magnitudes are processed by different systems/mechanisms and that numerical values are automatically processed, we would expect that: (1) performance in the numerical dimension would comply with Weber's law regardless of task demands; (2) when the physical dimension is relevant, performance in the physical task might deviate from Weber's law; and (3) when the physical dimension is irrelevant (in the numerical task), performance in the physical dimension might not deviate from Weber's law due to an interaction with a very accurate numerical magnitude representation.

Questions regarding shared or separate representation of different magnitudes and the interactions between such representations are of great developmental interest. Odic et al. (2013) had 3- to 6-year-olds discriminate between quantities (numbers) or the area of irregular shapes. They found that the acuity of area discrimination was better than number discrimination. Namely, participants were able to detect much smaller changes in area than in numerosity. However, both area acuity and number acuity showed a similar growth function throughout development. Thus, the authors raised the possibility of similar yet separated development for different magnitudes. Similarly, Lourenco and Longo (2010) suggested that in early age, all magnitudes are represented by a shared system. However, with time, there is a division of this system into subsystems that specialize in the processing of specific magnitudes. Some theories suggest that understanding non-symbolic number is the basis for understanding symbolic numbers. Von Aster and Shalev (2007), for example, suggested a 4-step developmental model of numerical cognition. According to this model, we are born with the ability to represent and approximate the cardinality of magnitudes. This ability provides the basic meaning of numbers (step 1). In step 2, the child learns to associate quantity with number words, and in step 3, with the Arabic numeral symbols. The association to Arabic symbols is a precondition for the development of the mental number line (step 4). In step 4, ordinality is represented as a second (and acquired) core system for numbers.

The current experiment provides a picture about the representation of symbolic (numbers) and non-symbolic (physical size) magnitudes (Experiment 1), and the interaction between these representations (Experiment 2) in adults. This data and this methodology can serve as a baseline for studies aiming to investigate representation of symbolic and non-symbolic sizes (and an interaction between them) at different points in normal and impaired development.

## Experiment 1—uni-dimensional comparisons

### Methods

#### Participants

Fourteen volunteers (11 females, 3 male, mean age: 23 years), students from Ben-Gurion University of the Negev or Achva Academic College, participated in the experiment for class credit. All participants had intact or corrected vision and no learning disabilities. Seven performed the physical task first and seven performed the numerical task first.

#### Stimuli

Each stimulus was composed of two digits from 1 to 9. The numbers (Courier New font) appeared in lime color on a black background, each 1.75 cm from the center of a computer screen (i.e., center of the number to center of the screen). The participants sat at a distance of about 50 cm from the screen. Numbers (1–9) were paired to create eight numerical ratios (0.1–0.8). The ratios were rounded to one digit after the decimal point (see Table 1). Note that the ratio is a continuum. Thus, although the ratios in every category may differ from pair to pair, all the pairs with ratio 0.3, for example, are larger than 0.2 and smaller than 0.4 [see similar design in Cantlon and Brannon (2006)]. For example, to create the numerical ratio of 0.3, we used the pair 2 and 6 (2/6 = 0.3). Similarly, nine font sizes (12.5, 25, 37.5, 50, 62.5, 75, 87.5, 100, and 112.5) were paired in order to create eight physical size ratios (0.1–0.8). The physical sizes were adopted from Cohen Kadosh et al. (2008b)—Experiment 2. The pairs of physical sizes we used are outlined in Table 2. For example, to create the physical ratio of 0.5, we used the fonts 50 and 100 (50/100 = 0.5) or 25 and 62.5, or 37.5 and 75. The same sizes were used for more than one physical ratio to avoid confounding of size and ratio.

**Table 1:** **Pairs of stimuli by numerical ratio**.

**Category ratio**	**Ratio**	**Large number**	**Small number**
0.1	0.11	9	1
	0.13	8	1
	0.14	7	1
0.2	0.20	5	1
	0.22	9	2
	0.25	4	1
	0.25	8	2
0.3	0.33	3	1
	0.33	6	2
	0.33	9	3
0.4	0.40	5	2
	0.43	7	3
	0.44	9	4
0.5	0.50	2	1
	0.50	4	2
	0.50	6	3
	0.50	8	4
0.6	0.60	5	3
	0.63	8	5
	0.67	3	2
	0.67	6	4
	0.67	9	6
0.7	0.71	7	5
	0.75	4	3
	0.75	8	6
0.8	0.80	5	4
	0.83	6	5
	0.86	7	6

*Ratio, (small number/large number) with an accuracy of 2 decimal places*.

**Table 2:** **Pairs of stimuli by physical ratio**.

**Category ratio**	**Ratio**	**Large font size**	**Small font size**
0.1	0.11	112.5	12.5
	0.13	100	12.5
	0.14	87.5	12.5
0.2	0.20	62.5	12.5
	0.22	112.5	25
	0.25	50	12.5
	0.25	100	25
0.3	0.33	37.5	12.5
	0.33	75	25
	0.33	112.5	37.5
0.4	0.40	62.5	25
	0.43	87.5	37.5
	0.44	112.5	50
0.5	0.50	25	12.5
	0.50	50	25
	0.50	75	37.5
	0.50	100	62.5
0.6	0.60	62.5	37.5
	0.63	100	62.5
	0.67	37.5	25
	0.67	75	50
	0.67	112.5	75
0.7	0.71	87.5	62.5
	0.75	50	37.5
	0.75	100	75
0.8	0.80	62.5	50
	0.83	75	62.5
	0.86	87.5	75

*Ratio, (small size/large size) with an accuracy of 2 decimal places*.

Experiment 1 included three physical blocks and three numerical blocks. In the physical block, the same number appeared twice in different physical sizes. In total, a physical block contained 96 stimuli: 8 physical ratios (0.1–0.8) × 2 sides (larger number on left vs. on right) × 6 pairs of numbers. In the numerical block, different numbers appeared in the same physical size. In total, a numerical block contained 96 stimuli: 8 numerical ratios (0.1–0.8) × 2 sides (larger number on left vs. on right) × 6 pairs of numbers. In both tasks, the specific pairs of numbers and their specific physical sizes within a given ratio were randomly selected for every participant.

#### Procedure

**Figure 1:**
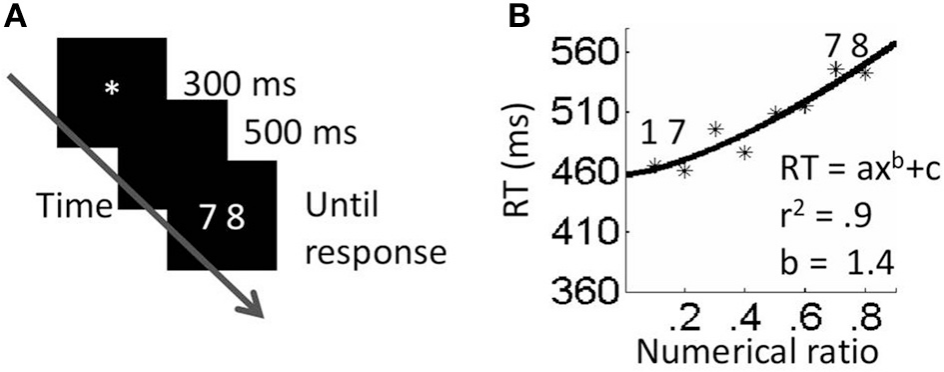
**Neutral numerical task. (A)** Procedure. **(B)** Results. Fitting the data to a power function and a linear function was not significantly different.

**Figure 2:**
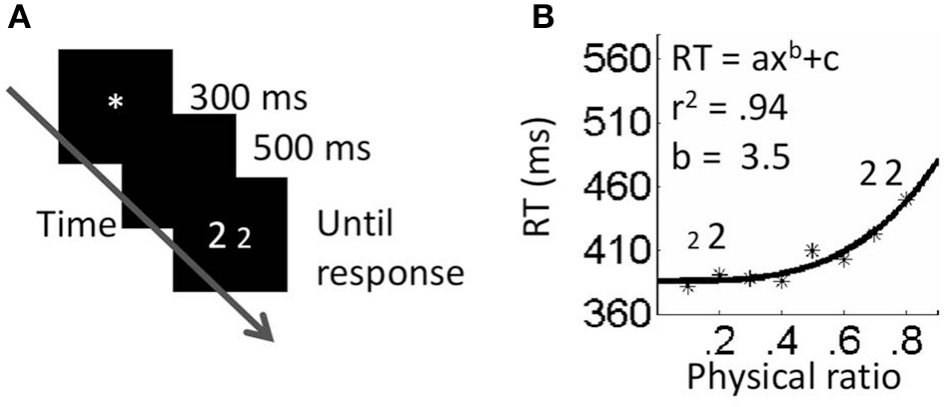
**Neutral physical task. (A)** Procedure. **(B)** Results. Fitting the data to a power function resulted in higher fits than fitting to a linear function.

Participants were asked to decide, as quickly as possible while avoiding errors, which of the two numbers was physically larger (in the physical block), or numerically larger (in the numerical block). They were asked to indicate their decision by pressing a key (*p* or *q*) corresponding to the side of the larger number. Each trial began with a central fixation point presented for 300 ms. Five hundred ms after the elimination of the fixation point, a pair of numbers appeared and remained in view until the participant pressed a key. The next trial started 500 ms after response onset (see Figures 1A, 2A). For every task, instructions and six practice trials were presented first, followed by three experimental blocks. The stimuli within a block appeared in a random order.

#### Design

For each task there was a single independent variable of ratio that had 8 possible values. The dependent measures were RT and accuracy.

### Results

We calculated error rates and mean RT in milliseconds (ms) for correct responses only, for every ratio (numerical and physical). Very low (less than 150 ms) and very high (over 3000 ms) RTs were excluded from the analysis (1 trial in the numerical task). These mean RTs were subjected to a One-Way analysis of variance (ANOVA) with ratio as an independent variable. The main effects of numerical and physical ratios were significant [(*F*_(7, 91)_ = 32.49, *MSE* = 465, *p* < 0.001, η^2^_*p*_ = 0.71) and (*F*_(7, 91)_ = 18.92, *MSE* = 397, *p* < 0.001, η^2^_*p*_ = 0.59), respectively]. Namely, RT increased with magnitude ratio. Accuracy for the numerical task (mean = 0.97, *SD* = 0.16) and for the physical task (mean = 0.98, *SD* = 0.14) presented a similar pattern: for the numerical task: *F*_(7, 91)_ = 25.7, *MSE* = 0.0003, *p* < 0.001, η^2^_*p*_ = 0.66, and for the physical task: *F*_(7, 91)_ = 13.84, *MSE* = 0.0004, *p* < 0.001, η^2^_*p*_ = 0.52.

#### Exponents analyses

In order to investigate whether numerical and physical comparisons result in different functions, we plotted each participant's RT as a function of the magnitude ratio (i.e., physical ratio or numerical ratio) and fitted the plots to a power function (*RT* = *ax*^*b*^ + *c*) using the Matlab curve fitting tool. Then, the exponent values were used as a dependent variable in a *t*-test for dependent samples. Three participants were removed from this analysis due to *r*^2^ that deviated from the average by more than two statistical deviations. According to the results, the difference between the exponents for physical comparisons and numerical comparisons was significant. Specifically, the exponent was higher for the physical task (average exponent = 3.46, *SD* = 2.2) than for the numerical task (average exponent = 1.44, *SD* = 0.75) *t*_(10)_ = 2.92, *p* < 0.05. A one-sample *t*-test revealed that while the exponents in the physical task were significantly different than one [*t*_(10)_ = 3.4, *p* < 0.01], the exponents in the numerical task were not significantly different than one [*t*_(10)_ = 1.71, *ns*]. This suggests that performance in the physical comparison task, but not the numerical comparison task, deviated from linearity, thus violating Weber's law.

To further strengthen this suggestion, we fitted the data of every participant in every task twice; once to a linear function and once to a power function. We then used the fit values (*r*^2^) as the dependent measures in *t*-tests for dependent samples. This was done for each task separately. For the numerical task, there was no significant difference between the fits of the two functions (*t* < 1, *ns*). This was expected since in this task the exponent values calculated by the fitting process were close to one; this means that, in practice, the exponential equation behaved as a linear one. For the physical task, *r*^2^ values were higher when the data was fitted to a power function than when fitted to a linear function *t*_(12)_ = 2.9, *p* = 0.01.

### Discussion

The results of Experiment 1 revealed a different relationship between the RT and magnitude ratio for physical sizes and numerical values and suggest that the two dimensions have different representations. In the numerical task, performance complied with Weber's law. Namely, discriminability increased linearly. In contrast, in the physical task RT did not change by a constant amount, violating Weber's law; rather, for a fixed increment in size ratio, RT increased with the similarity between the stimuli, although not linearly.

We suggest that the difference between the representations (numerical and physical properties) might stem from the different nature of the stimuli: numbers are a special kind of magnitude: they are discrete, countable, symbolic representations with verbal labels. Physical sizes, on the other hand, are non-countable, continuous magnitudes that one can only estimate. Thus, while numbers are represented on a “mental *number* line” that complies with Weber's law, physical sizes may be represented on a more general “mental *magnitude* line” that is noisier due to the nature of continuous properties. Our data cannot determine between the possibility of one mental magnitude line with different levels of noise for different representations, or two separate systems: one that represents numbers, and one that represents continuous properties [similar to the suggestions of Odic et al. (2013) and Lourenco and Longo (2010)].

The current study involved adults. It will be interesting to use this design with children who are just starting to learn the numerical symbols system; if our hypothesis is correct and the linear representation is due to an exact verbal representation of numbers and the difference between them, then what trend will children early in their formal education produce? Studying this trend, and not only the existence of a ratio effect, can be more informative and detect more subtle changes in performance.

## Experiment 2—numerical stroop tasks

Given the congruity effect and the difference between the representations of symbolic and non-symbolic dimensions observed in Experiment 1, it is interesting to ask what happens to these representations in a numerical Stroop task, like the one employed by Henik and Tzelgov (1982). Can the relevancy of a dimension modulate mental representations? Can different representations co-exist? The following experiment addresses these questions.

### Methods

#### Participants

Twenty volunteers (15 females, 5 males, mean age: 22.95 years), students from Ben-Gurion University of the Negev or Achva Academic College, participated in the experiment for class credit. All participants had intact or corrected vision, and no learning disabilities. Ten participants performed the physical task and 10 performed the numerical task.

#### Stimuli

**Figure 3:**
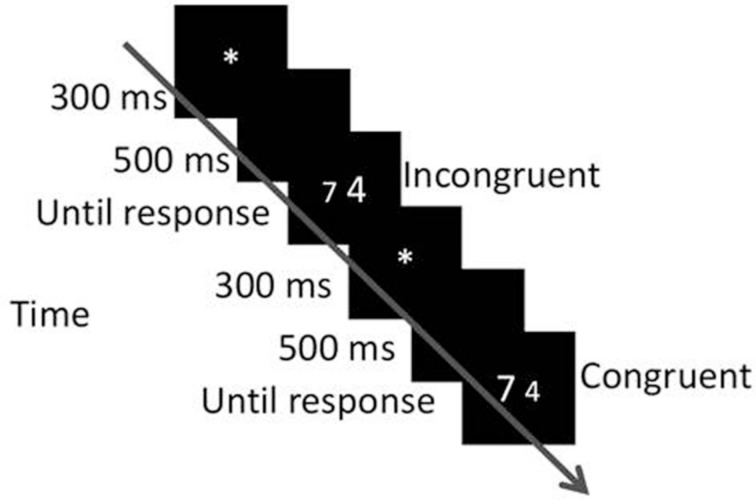
**Procedure of Experiment 2.** Trials in these blocks were either congruent or incongruent.

The same physical sizes and numerical ratios of digits from Experiment 1 were used here. The stimuli created two congruency conditions: congruent or incongruent, as described by Henik and Tzelgov (1982) (see Figure 3). Similar to Experiment 1, instead of manipulating the distance between the numbers, we manipulated the ratio of both dimensions. We used eight physical ratios and eight numerical ratios. An experimental block (numerical block as well as physical block) contained 256 stimuli: 2 conditions (congruent, incongruent) × 8 physical ratios (0.1–0.8) × 8 numerical ratios (0.1–0.8) × 2 sides of presentation. The block repeated 14 times, 7 times in a session. In every block, the specific numbers and their absolute size were randomly selected.

#### Procedure

The procedure was similar to that of Experiment 1 except that blocks included congruent and incongruent trials, as can be seen in Figure 3. The experiment was completed in two sessions.

### Results

In the physical task, the average accuracy rate was 0.97 (*SD* = 0.17), and in the numerical task, the average accuracy rate was 0.95 (*SD* = 0.21). In both tasks, there was not enough variance to analyze accuracy rates for the different conditions.

Mean RT in milliseconds was calculated for correct responses only. Very high (over 3000 ms) and very low (under 150 ms) RTs were eliminated from the analysis (3 trials—only in the numerical task). Mean RTs were subjected to a three-way ANOVA with physical size ratio (0.1–0.8), numerical size ratio (0.1–0.8) and congruity (congruent and incongruent) as independent variables. Physical and numerical tasks were analyzed separately.

**Figure 4:**
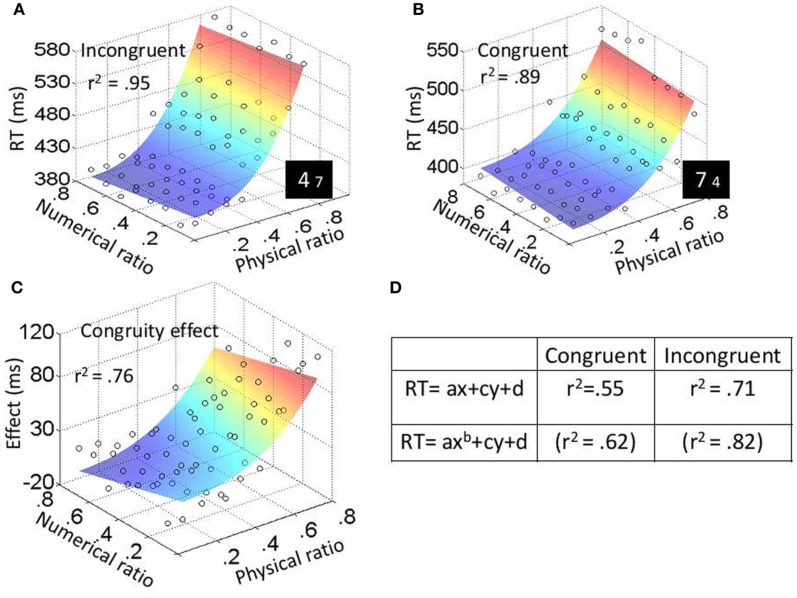
**Experiment 2: physical task results.** Average RT (for every participant in every condition) was plotted as a function of both physical (*x*-axis) and numerical (*y*-axis) ratios. The color of the surface represent RTs—blue is for the lowest RTs and red for the highest RTs. Fit values for plots **(A)**, **(B)**, and **(C)** are higher than the average of the fits based on individual participants. **(D)** Fits (*r*^2^) when fitting the plots of the different conditions to function (1) or function (2).

In the physical task, the three main effects were significant: physical ratio, *F*_(7, 63)_ = 103.12, *MSE* = 4472, *p* < 0.001, η^2^_*p*_ = 0.92; numerical ratio, *F*_(7, 63)_ = 4.04, *MSE* = 398, *p* < 0.001, η^2^_*p*_ = 0.31; and congruity, *F*_(1, 9)_ = 64.88, *MSE* = 2560, *p* < 0.001, η^2^_*p*_ = 0.88. The effect of the physical and the numerical ratios can be seen in Figures 4A,B; RT was the slowest when the ratio between the physical magnitudes (the relevant dimension) was 0.8 (the smallest difference), and the ratio between the numerical values (the irrelevant dimension) was 0.1 (the largest difference). Physical ratio was found to influence congruity [*F*_(7, 63)_ = 18.8, *MSE* = 1374, *p* < 0.001, η^2^_*p*_ = 0.68] as did numerical ratio [*F*_(7, 63)_ = 6.4, *MSE* = 729, *p* < 0.001, η^2^_*p*_ = 0.42]. As can be seen in Figure 4C, the congruity effect was the strongest when the ratio between the physical magnitudes was 0.8, and the ratio between the numerical values was 0.1. The triple interaction between physical ratio, numerical ratio and congruity was not significant [*F*_(49, 441)_ = 1.2, *ns*, η^2^_*p*_ = 0.12].

**Figure 5:**
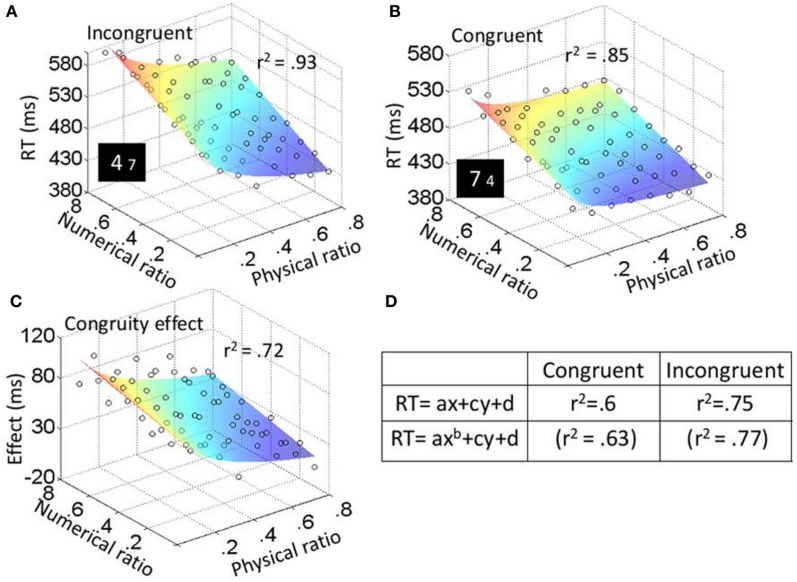
**Experiment 2: numerical task results.** Average RT (for every participant in every condition) was plotted as a function of both physical (*x*-axis) and numerical (*y*-axis) ratios. The color of the surface represent RTs—blue is for the lowest RTs and red for the highest RTs. Fit values for plots **(A)**, **(B)**, and **(C)** are higher than the average of the fits based on individual participants. **(D)** Fits (*r*^2^) when fitting the plots of the different conditions to function (1) or function (2).

In the numerical task, the three main effects were significant: physical ratio, *F*_(7, 63)_ = 67.08, *MSE* = 1250, *p* < 0.001, η^2^_*p*_ = 0.88; numerical ratio, *F*_(7, 63)_ = 113.15, *MSE* = 977, *p* < 0.001, η^2^_*p*_ = 0.93; and congruity, *F*_(1, 9)_ = 78.11, *MSE* = 8795, *p* < 0.001, η^2^_*p*_ = 0.9. The effect of the physical and the numerical ratios can be seen in Figures 5A,B; RT was the slowest when the ratio between the numerical values (the relevant dimension) was 0.8 (the smallest difference), and the ratio between the physical sizes (the irrelevant dimension) was 0.1 (the largest difference). Physical ratio was found to influence congruity [*F*_(7, 63)_ = 19.61, *MSE* = 808, *p* < 0.001, η^2^_*p*_ = 0.69] as did numerical ratio [*F*_(7, 63)_ = 13.43, *MSE* = 410, *p* < 0.001, η^2^_*p*_ = 0.6]; this can be seen in Figure 5C. The congruity effect was strongest when the ratio between the numerical values was 0.8, and the ratio between the physical sizes was 0.1. The triple interaction between physical ratio, numerical ratio and congruity was not significant [*F*_(49, 441)_ = 1.2, *ns*, η^2^_*p*_ = 0.12].

#### Trend analyses

Similar to the previous experiment, we fitted the data to two functions:

(1) RT=ax + cy + d.

(2) $$RT=ax^{b}+cy+d.$$

In these functions, *x* represents the physical dimension (e.g., physical ratios from 0.1 to 0.8) and *y* represents the numerical dimension (e.g., numerical ratios from 0.1 to 0.8); *d* indicates the minimal RT; and *b* in function (2) is the exponent of the physical dimension. These functions are derived from the ANOVA main effects for physical (*x*) and numerical (*y*) ratios. The absence of a combined *xy* component in the functions reflects the lack of interaction between these two dimensions. According to our results from Experiment 1, function (1) is expected to give the best fit according to Weber's law (i.e., should fit to the results of the numerical task of Experiment 2). On the other hand, the best fit for the physical task of Experiment 2 should be function (2) since the numerical dimension was linear while the physical dimension deviated from linearity. Similar to Experiment 1, we fitted the plot for every participant in every task (physical or numerical) and every condition (congruent and incongruent) and recorded the fit (*r*^2^) values (see Figures 4D, 5D). These fits were then used as dependent variables in a two-way ANOVA, with task as a between-subject variable and condition as within-subject variable. For both the physical and numerical tasks, fits were higher for function (2) [*F*_(1, 18)_ = 38.1, *MSE* = 0.002, *p* < 0.001, η^2^_*p*_ = 0.38]. The analysis also revealed a main effect for condition, where fits were higher for incongruent than for congruent trials [*F*_(1, 18)_ = 45.6, *MSE* = 0.01, *p* < 0.001, η^2^_*p*_ = 0.72]. In addition, a significant Two-Way interaction between task and condition was found; the difference between the fits for the numerical task was smaller than for the physical task [*F*_(1, 18)_ = 5.97, *MSE* = 0.002, *p* < 0.05, η^2^_*p*_ = 0.25].

## General discussion

In the current study we took advantage of the fact that: (1) visually presented numbers have two dimensions of magnitude; and (2) that numbers are processed automatically, to answer two main questions. First, is there a difference between the representations of numerical and physical magnitudes? Second, can the relevancy of the dimension change its representation? To answer these questions, participants compared the physical size or the numerical values of pairs of numbers. In Experiment 1, only one dimension was manipulated. In Experiment 2, both dimensions were orthogonally manipulated to create congruent and incongruent conditions.

In Experiment 1, we found that performance in the numerical task fits the notion of Weber's law, as predicted by the literature. In contrast, performance in the physical task, though still ratio-dependent, deviated from Weber's law. This was evidenced by two results. First, fits for power functions were higher than for linear functions only in the physical task. Second, when fitting the plots to an exponential function, the exponents obtained in the physical task were significantly higher than those obtained in the numerical task. This pattern of results fits our suggestion that differences exist between representations of numerical and physical magnitudes. We hypothesized that these differences could be attributed to the exact verbal representation of numerical values in comparison to the less accurate nature of physical sizes. However, more research is necessary to confirm this hypothesis. One way to further test this hypothesis is through a developmental study with children in different stages of their familiarity with the numerical symbols systems. If our hypothesis is correct, plotting RT as a function of the numerical ratio, and fitting this plot to a power function, will yield an exponent greater than 1. This exponent value will become similar to 1 when the child gains experience with the numerical symbols system.

Our suggestion is in line with other findings in the literature connecting exact representation with magnitude representation. Whalen and colleagues (1999) presented participants with a target number and asked them to press a key repeatedly (without counting) until they believed that they reached the target number. These results complied with the results of a similar experiment with animals—the number of presses increased with the target number, suggesting scalar variability (i.e., encoding of magnitudes is noisy, and this noise increases proportionally with magnitude) (Gallistel and Gelman, 2000). In a similar design, participants had to reproduce time durations. The coefficient of the variance (the ratio between the variability of the estimation and its average) was higher for estimation of time duration than for non-verbal counting. Duration of time is continuous, while key pressing is discrete. This may be related to the change in the coefficient of variance in the two tasks. In a similar study, Cordes et al. (2001) conducted a key-press experiment with adults under conditions that required either counting aloud or did not allow vocal or sub-vocal counting (i.e., allowed only non-verbal counting). In the non-verbal counting condition, the authors found a power law relationship between the target number and the average number of presses, suggesting that alongside of the non-verbal counting mechanisms that we share with other animals, there exists another representation for verbal counting.

Additional evidence for the influence of semantic meaning on the representation of the mental number line comes from line-mapping experiments with children. Ebersbach et al. (2008) asked children between the ages of 5- to 9-years old to map numbers onto a number line. They found that the representation of the mental number line was influenced by familiarity; mapping was linear for familiar numbers and logarithmic for less familiar numbers. Similar results were obtained in a study by Siegler and Opfer (2003). In this study, 7-year-olds or adults had to map numbers onto a line from 0 to 100 or 0 to 1000. While both children and adults revealed the same linear mapping for 0–100. Children, who were unfamiliar with numbers above 100, mapped the numbers logarithmically when they were beyond 100.

Experiment 2 included numerical and physical Stroop tasks. Unlike previous studies, we had 8 physical and 8 numerical ratios, and plotted RT as a function of both the physical and the numerical ratio. In that way, we were able to analyze differences in the trends of the different dimensions. Our results suggested that the representation of the physical dimension depends on task demands; when the physical dimension was relevant its trend was exponential, similar to the trend of the neutral task (in Experiment 1). As a result, there was a large difference between the fits to function (1) that assumes linearity for both physical and numerical dimensions, and function (2) that allows deviation from linearity for the physical dimension. Namely, fits were higher for function (2). In contrast, when the numerical dimension was relevant, there was a very small difference between the fits to functions (1) and (2). This suggests that the trend of the physical dimension shifted and became more “linear.”

We propose that this shift of exponents is due to an interaction between the exact magnitude representations of the numerical values with the estimated magnitude representation of physical continuous dimensions. Specifically, we suggest that when participants were asked about the physical size of the numbers, they activated a spontaneous mental *magnitude* line, which is noisier and less organized than the exact number line (Izard and Dehaene, 2008). The result of such spontaneous activation of the mental *magnitude* line representation is the deviation from linearity, much as in the neutral task in Experiment 1. In contrast, when asked about the numerical value of the numbers, participants activated an exact mental number line representation. The physical sizes, in turn, could have been mapped onto that line. To confirm our suggestion, one can change the stimuli used in the experiment. For example, compare two continuous magnitudes—brightness and area of squares. According to our hypothesis, since both dimensions are continuous, their trend should be exponential, regardless of the irrelevant dimensions. Since RT was faster and accuracy was higher in the physical tasks, there is a possibility that the deviation from linearity was a result of a floor effect in small ratios, where the task was very easy to perform. This is a built-in limitation: we found here that participants were much faster in comparing sizes than comparing numbers, when comparing a wide range of ratios. This alone provides important information about the processing mechanism—something in the processing of the physical size allows it to be faster and more accurate, and to deviate from Weber's law. If we try to artificially encourage slower RT, it will no longer be comparable to the discrete task.

Our experimental design and analysis provide a tool that developmental studies in the field of numerical cognition can benefit from for several reasons. First, changes throughout development might be subtle. Using a wide range of ratios, instead of 2 or 3 ratios, and analyzing the function created when RT is plotted as a function of magnitude ratio, might uncover differences that would be missed in the commonly studied age × (2–3 points) ratio interaction. The analysis of Experiment 1 can be used to ask how different magnitudes are represented at different stages of development. The analysis used in Experiment 2—analyzing the effect of both physical and numerical ratios on performance—can answer questions regarding an interaction between representation of numbers and physical sizes at different ages.

In conclusion, in the current work we examined the ratio effect in finer resolution compared with studies reported so far in order to detect differences between representation of numerical values and physical sizes. To the best of our knowledge, this is the first work in numerical cognition to use the exponent as a dependent variable and to investigate the combined influence of different magnitude ratios on RT in a comparative judgment task.

Our results, coupled with the current literature, suggest that numerical values and physical magnitudes have different representations. This can be the result of two different yet interacting core systems: a core system that represents continuous magnitudes, and a system that represents discrete magnitudes. These systems are shared across species. The system for continuous magnitudes is ratio-dependent but does not necessarily comply with Weber's law. Our pattern of results is in favor of a previous suggestion that the system for processing continuous magnitudes might be older than the system for processing discrete magnitudes (Cantlon et al., 2009; Henik et al., 2012), although further research is needed to support this notion. An interaction between symbolic processes (language) and a system for representation of discrete magnitudes may explain the special and exact representation of numbers, as supported by the developmental studies mentioned above. The interaction between the continuous and discrete representations is manifested in a change of trend of the physical dimension when the numerical dimension is relevant; activating the mental number line to resolve a numerical Stroop task allows for a less exponential representation of the irrelevant physical dimension. Note that the representation of continuous magnitudes on a mental magnitude line has been less investigated, and it is hard to hypothesize how some of the current models apply to continuous magnitudes. For example, Verguts et al. (2005) suggested that the representation of different quantities is described by a place-coding. It is hard to understand how the concept of continuous magnitudes can be described by a place-coding. Thus, more studies in the field are required to confirm our suggestion.

### Conflict of interest statement

The authors declare that the research was conducted in the absence of any commercial or financial relationships that could be construed as a potential conflict of interest.

## Footnotes

^1^“Exact representation” refers to a specific value that goes with the numerical symbol (i.e., 3 represents exactly 3 items). This is in contrast to physical sizes, such as length, that do not have verbal labels to represent them, and which we can only estimate their exact size. Both exact and non-exact magnitudes can be placed on a magnitude line, which is logarithmic with overlapping representations. However, as suggested by Izard and Dehaene (2008), being able to represent exact and not estimated size, might “calibrate” the mental number line. Namely, we still expect a ratio effect but this ratio effect might have different manifestations (such as different exponents).
